# Combined petrosal approach for a huge retroclival meningioma preserving the cranial nerves

**DOI:** 10.3171/2022.1.FOCVID21221

**Published:** 2022-04-01

**Authors:** Dong-Won Shin, Chang-Ki Hong

**Affiliations:** Department of Neurosurgery, Asan Medical Center, College of Medicine, University of Ulsan, Seoul, Republic of Korea

**Keywords:** meningioma, skull base, cranial nerves, microsurgery

## Abstract

Surgery for petroclival meningioma is challenging because cranial nerve preservation during tumor removal can be very complex. For small- to medium-sized tumors, the anatomical relationship between tumor and neurovascular structures can be assessed before surgery. However, in large tumors, cranial nerves usually cannot be seen in preoperative images. The authors present a case of a 65-year-old woman who presented with gait disturbance and hearing loss and was diagnosed with huge retroclival meningioma involving the cavernous sinus, Meckel’s cave, and internal acoustic meatus. In this video, they explain the radiographical, anatomical, and surgical considerations and demonstrate the surgical technique.

The video can be found here: https://stream.cadmore.media/r10.3171/2022.1.FOCVID21221

## Transcript

This video will demonstrate the microsurgical resection of a huge petroclival meningioma with preservation of the cranial nerves.

### 0:29 Patient Information.

The patient is a 65-year-old female who presented with gait disturbance.

### 0:35 Physical Examination.

Her physical examination revealed right-side hearing loss, but no other neurological symptoms were observed.

### 0:41 Preoperative Imaging.

Magnetic resonance imaging (MRI) demonstrated a well-enhanced mass occupying the cavernous sinus with extension to the clivus, internal auditory canal, and Meckel’s cave. Proton density enhancement MRI usually indicates the location of the cranial nerves as shown in the left-side MRI of another patient. However, in this case, we can only identify both optic nerves and facial nerve complexes before surgery.

### 1:10 Approaches.

It was difficult to fully expose the tumor with one approach alone, such as the anterior petrosal, posterior petrosal, or retrosigmoid approach. The posterior petrosal or retrosigmoid approach would provide only a limited surgical view of the tumor above the Meckel’s cave or crossing the midline. Meanwhile, the anterior petrosal approach alone would show a limited surgical view of the tumor below the internal acoustic meatus, though we may extend drilling to the rhomboid fossa. We considered the combined petrosal approach the most appropriate approach for this case.

### 1:47 Craniotomy Simulation.

As you can see in this real surgical view, the tumor is surrounded by the clivus, brainstem, and petrous portion of the temporal bone. When we simulate posterior petrosectomy and expose the Trautmann triangle, we can see that the anterolateral portion of the tumor is not exposed. Conversely, if we perform only anterior petrosectomy, the posterolateral portion of the tumor is not exposed. We thought that combined, the petrosal approach can provide the optimal surgical corridor for this case. Additionally, the retrosigmoid approach is not a good choice for this tumor because of the limited unilateral view, as shown here.

### 2:32 Position and Skin Incision.

The patient was in the three-quarter position, and the head was rotated 90° to the contralateral side. We prefer an inverted U-shape skin incision for the combined petrosal approach. Motor, somatosensory evoked potential (MEP and SSEP), facial nerve EMG, facial MEP, and auditory brainstem responses are monitored. We performed the whole procedure in 1 day.

### 2:56 Posterior Petrosal Approach.

Mastoidectomy was performed by the ENT department. The mastoid antrum and facial nerve were identified. Because the patient had right-side hearing loss and the tumor crossed the midline of the clivus, the translabyrinthine approach was preferred.

### 3:23 Anterior Petrosal Approach.

The dura propria of the greater superficial petrosal nerve and the V3 was sharply dissected. Using a cutting burr, a premeatal triangle was drilled, followed by removal of the postmeatal triangle and petrous ridge. Then, a diamond burr was used to avoid injury of the posterior fossa dura and inferior petrosal sinus.

### 3:51 Dura Incision.

The presigmoid dura was incised parallel to the sigmoid sinus as well as the temporal lobe dura. The superior petrosal sinus was carefully ligated and cut. It is essential to identify the location of the trochlear nerve before cutting the tentorium, which is usually deviated by the tumor.

### 4:15 Tumor Exposure and Removal.

After cutting the tentorial edge, the trochlear nerve was identified at the lateral side of the tumor. We also opened the porus trigeminus to remove tumor tissue from inside Meckel’s cave. It is essential to remove the tumor in Meckel’s cave because trigeminal symptoms would not be relieved until we decompressed the tumor here. The trigeminal nerve was deviated to lateral side of the tumor in this case, which implied this was true petroclival meningioma case. We can see the brainstem at the medial side of the tumor with the clear dissection plane. Tumor debulking helped to increase the working space. The oculomotor nerve was identified at the superior pole of the tumor. The ipsilateral posterior cerebellar artery was carefully dissected and detached from the tumor. The basilar bifurcation was easily identified by following the ipsilateral posterior cerebellar artery. We usually pushed Surgicel between the tumor and normal structures and then created a space with cottonoid patties. Three-hands technique is useful when we remove the tumor adjacent to neurovascular structure. Tumor decompression and dissection were conducted between the critical vascular structures and nerves. Tumor debulking with ultrasonoaspirator and piecemeal removal are mandatory to secure surgical working space. The ipsilateral abducens nerve was identified. The contralateral facial nerve was finally identified after we removed all tumor tissue. Residual tumor tissue at the clivus and contralateral side were also removed. The bilateral trigeminal nerves were microscopically observed because the large tumor had occupied the retroclival space. Both oculomotor nerves and posterior communicating arteries were also identified. The ipsilateral trigeminal, facial, and lower cranial nerves were well preserved without significant injury. Reconstruction was performed with an artificial dura substitute, fibrin sealant patch, and fibrin glue. Lumbar drain was maintained for 3 days after surgery to prevent CSF leakage.

### 7:53 Postoperative MRI.

Postoperative MRI revealed near-total resection. Small residual tumor tissue was left at the right cavernous sinus and the clivus. The patient had oculomotor nerve paralysis immediately after surgery but recovered after 6 months. Trochlear nerve palsy did not improve, so she underwent surgery in the ophthalmological department. Facial palsy was not observed, and the other cranial nerves were neurologically intact.

### 8:22 Conclusions.

Surgical resection of petroclival meningioma is challenging regardless of the tumor size. For cases of small- to medium-sized tumors, we can localize the cranial nerves based on proton density gadolinium-enhanced MRI. However, for large tumors, it is difficult to preoperatively assess the tumor-nerve relationship. Kawase et al.^2,5^ described that there is no uniform location of cranial nerves in petroclival meningiomas. In these cases, we assess the location of the cranial nerves during surgery based on the tumor growth direction and site of origin. Petroclival meningiomas usually grow along the clivus, extend to the middle fossa through Meckel’s cave, or extend below the internal acoustic meatus. In each case, we carefully remove the tumor and avert the cranial nerves to preserve them.

### 9:17 References^1–6^

## Acknowledgments

Special thanks to the Department of Medical Contents, Asan Medical Center.

## Disclosures

The authors report no conflict of interest concerning the materials or methods used in this study or the findings specified in this publication.

## Author Contributions

Primary surgeon: Hong. Assistant surgeon: Shin. Editing and drafting the video and abstract: both authors. Critically revising the work: both authors. Reviewed submitted version of the work: both authors. Approved the final version of the work on behalf of both authors: Hong. Supervision: Hong.

## Supplemental Information

### Current Affiliations

Dr. Shin: Department of Neurosurgery, Gil Medical Center, Gachon University College of Medicine, Incheon, Republic of Korea.
